# Comparison of transcriptome between high- and low-marbling fineness in *longissimus thoracis* muscle of Korean cattle

**DOI:** 10.5713/ab.21.0150

**Published:** 2021-06-24

**Authors:** Seok-Hyeon Beak, Myunggi Baik

**Affiliations:** 1 Department of Agricultural Biotechnology and Research Institute of Agriculture and Life Sciences, College of Agriculture and Life Science, Seoul National University, Seoul 08826, Korea; 2 Institue of Green Bio Science and Technology, Seoul National University, Pyeongchang 25354, Korea

**Keywords:** Adipocytes, Computer Image Analysis, Intramuscular Fat, Korean Native Cattle, Marbling Fineness, Transcriptome

## Abstract

**Objective:**

This study compared differentially expressed genes (DEGs) between groups with high and low numbers of fine marbling particles (NFMP) in the *longissimus thoracis* muscle (LT) of Korean cattle to understand the molecular events associated with fine marbling particle formation.

**Methods:**

The size and distribution of marbling particles in the LT were assessed with a computer image analysis method. Based on the NFMP, 10 LT samples were selected and assigned to either high- (n = 5) or low- (n = 5) NFMP groups. Using RNA sequencing, LT transcriptomic profiles were compared between the high- and low-NFMP groups. DEGs were selected at p<0.05 and |fold change| >2 and subjected to functional annotation.

**Results:**

In total, 328 DEGs were identified, with 207 up-regulated and 121 down-regulated genes in the high-NFMP group. Pathway analysis of these DEGs revealed five significant (p<0.05) Kyoto encyclopedia of genes and genomes pathways; the significant terms included endocytosis (p = 0.023), protein processing in endoplasmic reticulum (p = 0.019), and adipocytokine signaling pathway (p = 0.024), which are thought to regulate adipocyte hypertrophy and hyperplasia. The expression of sirtuin4 (p<0.001) and insulin receptor substrate 2 (p = 0.043), which are associated with glucose uptake and adipocyte differentiation, was higher in the high-NFMP group than in the low-NFMP group.

**Conclusion:**

Transcriptome differences between the high- and low-NFMP groups suggest that pathways regulating adipocyte hyperplasia and hypertrophy are involved in the marbling fineness of the LT.

## INTRODUCTION

In beef, intramuscular fat (IMF) or marbling is associated with flavor, juiciness, and tenderness and is a critical factor determining the grade of beef in some countries, including Korea and Japan [1,2]. The IMF content is positively correlated with the beef price [3], and highly marbled beef is a premium consumer product, especially in Korea and Japan [4].

Computer image analysis (CIA) systems have been developed to assess various characteristics of marbling particles (MPs), such as their number, size, and distribution (fineness or coarseness) [5]. We recently reported that the number of fine MPs (NFMP) in the *longissimus thoracis* muscle (LT) was positively correlated with the auction price of Korean cattle beef [6], indicating that Korean consumers prefer beef with fine MPs (each covering an area of 0.01 to 0.5 cm^2^). The marbling fineness is determined by the marbling fineness index, which is obtained by dividing the NFMP by the ribeye area (cm^2^) and represents the distribution of fine MPs in the LT. A CIA method has been established for measuring fine MPs, but the mechanism of fine MP formation is unknown.

High-throughput RNA sequencing is a powerful tool for quantifying transcripts [7]. Several RNA sequencing studies have examined the molecular mechanisms of IMF deposition and identified differentially expressed genes (DEGs) between different IMF levels in the LT of cattle [8,9]. The molecular mechanisms responsible for marbling fineness are unknown. Therefore, this study identified DEGs between cattle with high and low NFMP in the LT to understand molecular events associated with formation of fine MPs.

## MATERIALS AND METHODS

### Animals

All experimental procedures followed the Animal Experimental Guidelines of the Seoul National University Institutional Animal Use and Care Committee and were approved by the committee (SNUIACUC: SNU-160408-4). We used LT samples from Korean cattle steers sampled in our previous study [6]. The details of the feeding trial, sampling, and CIA methods can be found in our previous study [6]. Briefly, the size and distribution of MPs were assessed using a CIA method in 43 Korean cattle steers. Of all LT samples, 10 were selected based on the NFMP and assigned to either a high- (n = 5) or low- (n = 5) NFMP group (Table 1). The NFMP was used as the selection criterion, because it is highly correlated with auction price of beef and is positively correlated with quality grade (R^2^ = 0.41, p<0.001) [6]. Three samples from each group were randomly selected for RNA sequencing analysis, and all 10 samples were used for quantitative real-time polymerase chain reaction (qPCR) analysis. (Table 1). Each three steers from the high and low group were randomly selected for RNA-seq, and total ten steers were used for qPCR to validate the result of RNA-seq.

### Carcass grading and the computer image analysis method

The carcass traits (back fat thickness, ribeye area, marbling score, and quality grade) were graded by trained meat graders according to the Korean carcass grading system of the Korea Institute for Animal Products Quality Evaluation [10]. The carcass auction price was determined by wholesalers after grading in the slaughter house. Detailed methods have been described previously [6].

Cross-sectional photographs of the ribeye area in the LT were taken alongside the 13th thoracic vertebra using HK-333 beef carcass photography equipment (mirror type) (Hayasaka Ricoh, Sapporo, Japan) developed by Kuchida et al [11], and the photographs were analyzed using Beef Analyzer II (Hayasaka Ricoh, Japan), as described previously [6,12]. CIA traits were obtained as described previously [6]. Briefly, the total number of MPs was counted from the binarized fat image. Fine MPs were defined as those covering an area between 0.01 and 0.5 cm^2^. The fineness index was obtained by dividing the NFMP by the ribeye area (cm^2^).

### RNA sequencing

Total RNA was extracted from LT tissue using TRIzol reagent (Sigma-Aldrich, Milwaukee, WI, USA) in accordance with the manufacturer’s protocol. Total RNA was quantified with a ND-2000 spectrophotometer (Thermo Fisher Scientific, Waltham, MA, USA) by recording the absorbance at 260 nm. The RNA quality was checked via agarose gel electrophoresis and ethidium bromide staining of the 28S and 18S bands, using an Agilent 2100 Bioanalyzer with the RNA 6000 Nano Chip (Agilent Technologies, Santa Clara, CA, USA). RNA samples were stored at −70°C until RNA sequencing and qPCR analyses. The cDNA library was constructed and sequenced at ebiogen (Seoul, Korea). The library was constructed using a SENSE 3′ mRNA-Sequencing Library Prep kit (Lexogen, Vienna, Austria) [13] according to the manufacturer’s instructions. Briefly, an oligo-dT primer containing an Illumina-compatible sequence at its 5′ end was hybridized with 500 ng of total RNA, and reverse transcription was initiated. After degrading the RNA template, the second strand was synthesized using a random primer containing an Illumina-compatible linker sequence at its 5′ end. To remove all reaction components, the double-stranded library was purified using magnetic beads. The library was then amplified to add the complete adapter sequences required for cluster generation. High-throughput sequencing was conducted as single-end 75-bp sequencing using a NextSeq 500 system (Illumina, San Diego, CA, USA). The quality of the sequencing reads from LT tissues was checked using FastQC. The reads that passed quality control were mapped to the *Bos taurus* genome (UMD3.1) from the UCSC database using Tophat2 (v2.0.2) and counted using HTSeq (v0.5.3p3).

### Identifying and annotating differentially expressed genes

For data analysis, the SENSE 3′ mRNA sequencing reads were aligned using Bowtie2 ver. 2.1.0. [14]. Bowtie2 indices were generated from either the genome assembly sequence or representative transcript sequences for alignment to the genome and transcriptome. The alignment file was used to assemble transcripts, estimate transcript abundances, and detect DEGs. DEGs between the high- and low-NFMP groups were determined based on counts from the unique and multiple alignments. The read count data were processed based on the quantile normalization method using Genowiz ver. 4.0.5.6 (Ocimum Biosolutions, Hyderabad, India). For functional annotation, we selected significant DEGs based on p<0.05 and |fold change (FC)| >2. The DEGs were categorized based on gene ontology (GO) and Kyoto encyclopedia of genes and genomes (KEGG) annotations using the Database for Annotation, Visualization and Integrated Discovery (DAVID) tool (v6.7) (http://david.abcc.ncifcrf.gov/) [15]. The RNA sequencing data obtained here have been submitted to the NCBI Gene Expression Omnibus (GEO) under accession number GSE166206 (http://www.ncbi.nlm.nih.gov/geo/).

### Quantitative real-time polymerase chain reaction

cDNA was synthesized from the RNA templates (2 μg) using an iScript cDNA synthesis kit (Bio-Rad Laboratories, Hercules, CA, USA). qPCR was performed as reported elsewhere [16] using QuantiTect SYBR Green RT-PCR Master Mix (QIAGEN, Hilden, Germany). Briefly, qPCR analyses were performed using Rotor-Gene Q (QIAGEN, Germany) in a 25-μL total reaction volume. The mixture contained 20 ng of cDNA, 1.25 μL of 10 μM primers, and 12.5 μL of SYBR Green RTPCR Master Mix. The reaction started at 95°C for 15 min, followed by 40 cycles of 94°C for 15 s, 55°C for 30 s, and 72°C for 30 s. Primer information can be found in Supplementary Table S1. The ΔΔCT method was used to determine relative FC values [17]. qPCR data were normalized relative to the level of the β-actin housekeeping gene, as validated in our previous study [16].

### Statistical analysis

Statistical differences between the two groups were determined using the independent t-test or Mann–Whitney test depending on data normality with IBM SPSS ver. 22 (SPSS, Chicago, IL, USA).

## RESULTS AND DISCUSSION

### Carcass and computer image analysis characteristics of the animals

We compared the carcass and CIA characteristics between the high- and low-NFMP samples used RNA sequencing and qPCR (Table 1). The high-NFMP group had a higher marbling score, NFMP, and fineness index than did the low-NFMP group (all p<0.001). The meat from the high-NFMP group also fetched a higher auction price (p<0.001), confirming the result in our previous report [6].

### RNA sequencing and identification of differentially expressed genes

**Figure 1:**
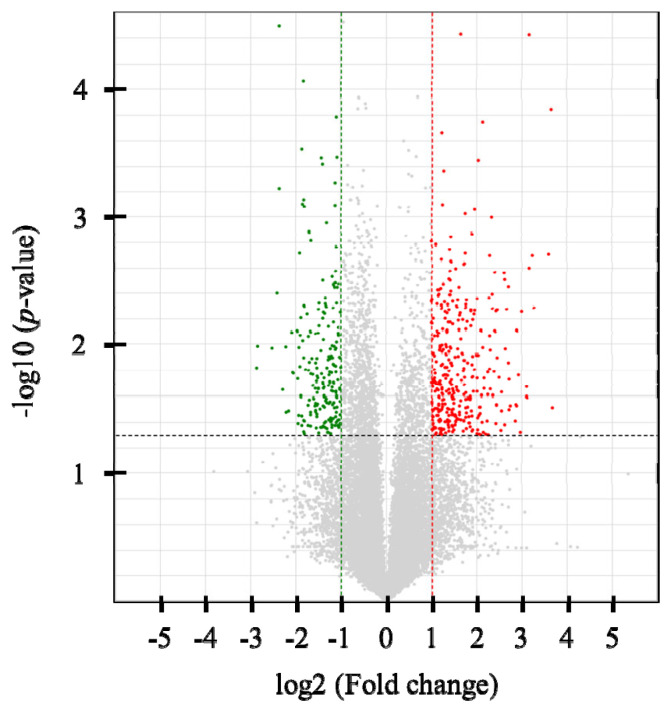
Volcano plot of RNA-sequencing data between the high and low NFMP groups. Up-regulated genes are colored red, and down-regulated genes are colored green in the high NFMP group. Two fold-up and down- regulated threshold lines were shown as dotted vertical red and green line, respectively, and p-value (<0.05) threshold line was shown as dotted horizontal line. NEMP, numbers of fine marbling particles.

Six cDNA libraries were constructed, and an average of 20,585,914 single-end 75-bp sequence reads was generated. At least 95.1% of the reads in each sample were mapped to the bovine reference genome (UMD3.1) using Tophat2 (v2.0.2) (Supplementary Table S2). In total, 328 DEGs were identified (p<0.05 and |FC| >2), with 207 up-regulated and 121 down-regulated genes in the high-NFMP group (Figure 1). The up- and down-regulated genes included 97 and 122 protein-coding genes, respectively. Table 2 lists the top 10 up- and down-regulated genes.

### Functional annotation of differentially expressed genes

The DAVID tool was used for functional annotation of the 328 DEGs, which were categorized as related to biological process, molecular function, and cellular component (Supplementary Tables S3 to S5). The number of genes associated with each pathway was small (Table 3). Nevertheless, there were significant pathway terms for DEGs, including endocytosis, protein processing in endoplasmic reticulum (ER), and adipocytokine signaling pathway, which are thought to affect adipocyte hypertrophy and hyperplasia.

Eight genes were found to be involved in the endocytosis pathway. Of these, three were up-regulated in the high-NFMP group: beta-arrestin-1 (*ARRB1*; FC = 2.07, p = 0.019), vacuolar protein sorting-associated protein 37D (*VPS37D*; FC = 2.54, p = 0.015), and ADP-ribosylation factor 3 (*ARF3*; FC = 2.35, p = 0.031). The other five genes involved in the endocytosis pathway in the high-NFMP group were down-regulated: ADP-ribosylation factor 4 (*ARF4*; FC = 0.47, p = 0.030), ARF-GAP with coiled-coil, ANK repeat and PH domain-containing protein 2 (*ACAP2*; FC = 0.31, p = 0.009), heat shock 70 kDa protein 1B (*HSPA1A*; FC = 0.36, p = 0.005), heat shock 70 kDa protein 6 (*HSPA6*; FC = 0.24, p = 0.008), and vacuolar protein sorting-associated protein 45 (*VPS45*; FC = 0.49, p = 0.013). Endocytosis is a vital cellular process in which extracellular ligands, soluble molecules, proteins, and lipids are taken up from the extracellular matrix [18]. Glucose transporter type 4 (GLUT4) is the most abundant glucose transporter regulated by insulin in adipocytes [19]. GLUT4 is translocated from the intracellular matrix to the plasma membrane or vice versa depending on insulin levels; this is orchestrated by exocytosis and endocytosis [20]. Both GLUT4 and CD36, an essential protein for fatty acid endocytosis and uptake in adipocytes, have similar trafficking and recycling systems induced by insulin [21]. Endocytosis is a crucial cellular process through which the uptake of glucose and fatty acid occurs. Through lipogenesis, adipocytes convert glucose into long-chain fatty acids and triglycerides, and finally store lipids as lipid droplets (LDs) [22]. Endocytosis related to GLUT4 and CD36 trafficking may lead to enlargement of the adipocytes (hypertrophy), which may in turn induce adjoining preadipocytes to proliferate (hyperplasia) by secreting paracrine growth factors [23]. Therefore, endocytosis may directly or indirectly affect marbling fineness.

All five DEGs involved in protein processing in the ER pathway were down-regulated in the high-NFMP group: DNAJ homolog subfamily A member 1 (DNAJA1; FC = 0.48, p = 0.003), DNAJ homolog subfamily B member 1 (DNAJB1; FC = 0.48, p = 0.011), endoplasmic reticulum oxidoreductase 1 beta (ERO1B; FC = 0.46, p = 0.042), HSPA1A, and HSPA6. The ER is a critical cellular organelle controlling calcium homeostasis, lipid synthesis, protein folding, and LD formation, and the size and number of LDs can be affected by the ER stress response [24]. The protein-folding or export capacity of the ER can be exceeded when the ER is stressed under some physiological and pathological conditions [25]. Several studies have reported LD accumulation [26], reduced adipocyte insulin sensitivity [27], and adiponectin oligomerization [28] during the ER stress response. Therefore, the involvement of ER protein processing in adipocyte hyperplasia and hypertrophy may contribute to marbling fineness.

All four DEGs involved in the adipocytokine signaling pathway were up-regulated in the high-NFMP group: long chain fatty acid CoA ligase ACSBG2 isoform X1 (ACSBG2; FC = 4.91, p = 0.002), 5′-AMP-activated protein kinase subunit gamma-2 isoform X1 (PRKAG2; FC = 2.15, p = 0.048), leptin receptor (LEPR; FC = 3.70, p = 0.007), and solute carrier family 2, facilitated glucose transporter member 1 (SLC2A1 or GLUT1; FC = 2.72, p = 0.019). Leptin is mainly secreted by mature adipocytes [29], and high adiposity and body fat increased the concentration and expression of leptin in cattle [30]. Leptin also can induce LD formation in several cell types in an mammalian target of rapamycin-dependent manner [31,32]. Genetically modified mice without the leptin receptor in adipose tissue were lighter than controls [33], and bone marrow stromal cells with impaired leptin receptors did not differentiate into adipocytes [34]. These results suggest that leptin can directly affect the development and function of adipocytes via leptin receptors. SLC2A1 is a class I glucose transporter that is expressed in adipose tissue at moderate levels [35]. PRKAG2 is a subunit of 5′-AMP-activated protein kinase [36]. Collectively, the adipocytokine signaling pathway may affect adipocyte hyperplasia or hypertrophy, possibly contributing to marbling fineness.

### Validation of differentially expressed genes using quantitative real-time polymerase chain reaction

To identify candidate genes associated with marbling fineness, we selected 10 DEGs which may be related with adipocyte hyperplasia and hypertrophy, although these were not listed in the top 10 up- and down-regulated genes shown in Table 2. Description of gene function in UniProt website was considered as selection standard (The UniProt Consortium [37]). The qPCR results were consistent with those from the RNA sequencing analysis for all genes tested, although statistical significance was not obtained in the qPCR analysis (Table 4).

We found higher sirtuin4 (SIRT4) expression in the high-NFMP group. Sirtuins are NAD+ dependent deacylases and ADP ribosyltransferases involved in cellular metabolism [38], and SIRT4 is a mammalian sirtuin (SIRT3, SIRT4, and SIRT5) located in mitochondria [39]. SIRT4 plays important roles in insulin secretion, glucose homeostasis [40], and lipid homeostasis, including fatty acid oxidation and lipid anabolism [41]. SIRT4 is highly expressed in bovine subcutaneous adipose tissue and regulates adipogenic differentiation-related marker genes, including transcription factor E2F1 and CCAAT/enhancer-binding protein β, during bovine adipocyte differentiation [40]. Overall, the up-regulation of SIRT4 expression may induce adipocyte differentiation, possibly contributing to increasing the NFMP.

We observed higher insulin receptor substrate 2 (IRS2) transcript levels in the LT samples from the high-NFMP group. IRS2 plays an important role in the metabolic activities of insulin and IGF1 in the liver, muscle, and adipose tissue [42]. Cell culture studies suggest that IRS2 also plays an important role in adipocyte differentiation [43]. Therefore, the up-regulated IRS2 expression in the high-NFMP group suggests that it is involved in the formation of MPs in the LT.

Several studies identified DEGs by RNA sequencing analysis and reported transcriptome or genes associated with the marbling score. For example, the DEGs from the contrast between age and marbling score were recently identified by RNA sequencing analysis in longissimus dorsi muscle of Korean cattle steers, and new potential early age markers and many genes for marbling development were found [44]. In this study, we compared DEGs between groups with high- and low- NFMP in the LT of Korean cattle, and the NFMP is used for calculation of fineness index. Our study has a novel aspect that we identified DEGs associated with marbling fineness, which is a new concept for marbling score.

## CONCLUSION

This study is a first attempt to understand the molecular events associated with the formation of fine MPs in the LT. Using RNA sequencing and KEGG pathway analyses, we found that three pathways (adipocytokine signaling pathway, endocytosis, and protein processing in the ER) involved with hyperplasia, hypertrophy, and adipocyte differentiation were significantly enriched in the DEGs. The *SIRT4* and *IRS2* genes, which are involved in adipocyte hyperplasia and hypertrophy, were up-regulated in the high-NFMP group. Our study suggests that pathways regulating adipocyte hyperplasia and hypertrophy are involved in marbling fineness. Further study is required to determine whether the balance between adipocyte hyperplasia and hypertrophy is linked to marbling fineness.

## Figures and Tables

**Table 1:** Carcass and CIA characteristics of all animal and animals used for RNA sequencing and qPCR in the high and low number of fine marbling particles groups

Variables	All animals^1)^ (n = 43)	Animals for qPCR				Animals for RNA sequencing			

		High NFMP (n = 5)	Low NFMP (n = 5)	SEM	p-value	High NFMP (n = 3)	Low NFMP (n = 3)	SEM	p-value
Carcass traits									
Carcass weight (kg)	425	429	420	10.5	0.688	437	425	12.6	0.677
Back fat thickness (mm)	13.6	15.4	10.2	1.64	0.116	11.0	10.7	0.79	0.859
Rib eye area (cm^2^)	90.8	93.8	86.6	3.54	0.175	97.3	89.0	2.18	0.030
Yield index^2)^	64.5	63.7	66.2	1.08	0.263	66.7	66.1	0.79	0.758
Marbling score^3)^	4.86	7.00	4.20	0.50	<0.001	7.33	4.00	0.76	<0.001
Auction price (₩)	17,593	20,938	17,706	595	<0.001	21,102	17,538	880	<0.001
CIA traits									
Number of MPs	3,085	3,519	2,399	381	0.151	4,042	2,560	602	0.257
NFMP	203	282	156	21.3	<0.001	289	158	29.9	<0.001
Fineness index	2.37	3.13	2.03	0.20	<0.001	3.10	1.98	0.26	<0.001

1) These data were reported in our previous study [6].

CIA, computer image analysis; qPCR, quantitative real-time polymerase chain reaction; NFMP, number of fine marbling particles; SEM, standard error of mean; MP, marbling particle.

2) Yield index = 68.184 − 0.625 × back-fat thickness + 0.13 × rib eye area − 0.024 × carcass weight + 3.23.

3) Marbling score: 1 = min; 9 = max.

**Table 2:** Top 10 up and down-regulated genes in the high number of fine marbling particles group

Order	Gene name	Gene symbol	Fold change (high/low)	p-value
Up-regulated				
1	ATCAY kinesin light chain interacting caytaxin	*ATCAY*	9.71	0.005
2	Solute carrier family 28 member 1	*SLC28A1*	9.45	0.002
3	BCL2 like 14	*BCL2L14*	8.98	0.003
4	TP53 target 5	*TP53TG5*	6.14	0.003
5	DEPP1 autophagy regulator	*DEPP1*	5.90	0.024
6	ATP binding cassette subfamily C member 6	*ABCC6*	5.79	0.046
7	Ankyrin repeat domain 13B	*ANKRD13B*	5.39	0.029
8	MAD2L1 binding protein	*MAD2L1BP*	5.36	0.041
9	Small G protein signaling modulator 2	*SGSM2*	5.30	0.003
10	Serine protease 12	*PRSS12*	5.16	0.025
Down-regulated				
1	Heat shock protein family A (Hsp70) member 6	*HSPA6*	0.24	0.008
2	SHC binding and spindle associated 1 like	*SHCBP1L*	0.27	0.009
3	Peroxisomal biogenesis factor 13	*PEX13*	0.27	0.036
4	Proteasome 26S subunit, non-ATPase 5	*PSMD5*	0.28	0.010
5	DTW domain containing 2	*DTWD2*	0.28	<0.001
6	Leucine rich repeat neuronal 4	*LRRN4*	0.28	0.047
7	Hepsin	*HPN*	0.28	0.001
8	Chromosome 7 open reading frame 57	*C4H7orf57*	0.30	0.042
9	Cytokine inducible SH2 containing protein	*CISH*	0.30	0.030
10	ArfGAP with coiled-coil, ankyrin repeat and PH domains 2	*ACAP2*	0.31	0.009

**Table 3:** Kyoto encyclopedia of genes and genomes pathway terms for differentially expressed genes

Term	Count	p-value	Genes
Legionellosis	5	<0.001	*GRO1, CXCL3, HSPA1A, HSPA6, ITGAM*
Protein processing in endoplasmic reticulum	5	0.019	*DNAJA1, DNAJB1, ERO1B, HSPA1A, HSPA6*
Endocytosis	8	0.023	*ARF3, ARF4, ACAP2, VPS37D, ARRB1, HSPA1A, HSPA6, VPS45*
Butanoate metabolism	3	0.027	*BDH1, AACS, ACSM2B*
Adipocytokine signaling pathway	4	0.027	*ACSBG2, PRKAG2, LEPR, SLC2A1*
Glycerophospholipid metabolism	4	0.059	*CDS1, CRLS1, ETNK2, GPD1L*
MAPK signaling pathway	5	0.083	*ARRB1, CACNB4, GADD45A, HSPA1A, HSPA6*
Toxoplasmosis	4	0.084	*ALOX5, HSPA1A, HSPA6*

**Table 4:** Comparison data of RNA sequencing and qPCR to validate the selected 10 differentially expressed genes between the high and low number of fine marbling particles groups

Gene name	Gene symbol	RNA-seq		qPCR	
	
		Fold change (high/low)	p-value	Fold change (high/low)	p-value
Zinc finger protein 354C	*ZNF354C*	0.32	0.008	0.61	0.440
Attractin	*ATRN*	0.46	0.003	0.45	0.220
Peroxisomal trans-2-enoyl-CoA reductase	*PECR*	0.46	0.003	0.50	0.260
Insulin receptor substrate 2	*IRS2*	2.05	0.043	1.64	0.150
C1q and TNF related 5	*C1QTNF5*	2.36	0.022	1.15	0.720
Peroxisome proliferator activated receptor delta	*PPARD*	2.41	0.003	1.60	0.370
Transforming growth factor beta induced	*TGFBI*	2.42	<0.001	1.44	0.400
Sirtuin 4	*SIRT4*	3.11	<0.001	1.86	0.080
Folliculin interacting protein 2	*FNIP2*	4.33	0.025	2.86	0.470
Solute carrier family 28 member 1	*SLC28A1*	9.45	0.002	1.10	0.740

qPCR, quantitative real-time polymerase chain reaction.

The qPCR results were consistent with those from the RNA sequencing analysis for all genes tested, although statistical significance was not obtained in the qPCR analysis.

## SUPPLEMENTARY MATERIAL

**Supplementary Table S1.** Primer sequences of selected DEGs for the qPCR **Supplementary Table S2.** The number of sequenced reads and percentage of aligned reads **Supplementary Table S3.** Significant biological process pathways of gene ontology analysis. **Supplementary Table S4.** Significant molecular function pathways of gene ontology analysis **Supplementary Table S5.** Significant cellular component pathways of gene ontology analysis
