# Supplementary material for: Comparison of transcriptome between high- and low-marbling fineness in longissimus thoracis muscle of Korean cattle
Source: Anim Biosci. 2021 Jun 24;35(2):196–203. doi: 10.5713/ab.21.0150 (PMC8738949; doi:10.5713/ab.21.0150)
Supplement: Supplementary file 1 [file ab-21-0150-suppl.pdf]

Table S1. Primer sequences of selected DEGs for the qPCR.

Gene symbol	Accession number	Primer	Sequence (5' - 3')	Product length (bp)
ZNF354C	XM_024995389.1	Forward	GATGCTTCAAGGGGAGTCGC	161
		Reverse	GAGCAGGCAGCAAATCCAAG	
ATRN	XM_025000260.1	Forward	ATTCACGGAGGCTACAAGGC	133
		Reverse	CAGCTGTGTGCAAGTAACGG	
PECR	XM_002685568.6	Forward	GTAATGTGGTCATTGCGTCCC	291
		Reverse	TGCACATATAGAAGGTGCCCG	
SMU1	NM_001038573.1	Forward	CCTCCATCTCGTCTCATGGC	116
		Reverse	TCTTTGACAGCTGCTTTGCC	
IRS2	XM_025000170.1	Forward	CAGCATCGACTTCTTGACGC	85
		Reverse	GTAGTCACAGGGATGGAGCC	
FRAS1	XM_024993475.1	Forward	CTTCTACCGTGTCCTTGGGC	100
		Reverse	AAGGTGGAAAGGTCAGTCCC	
PRKAG2	XM_024991251.1	Forward	AAAAACAGCAGCCAGAAGCG	125
		Reverse	CTGTCCACCTTACGAGACGG	
LSR	NM_001083394.1	Forward	GGCTTTTTCTGAGCACCTCG	99
		Reverse	GGGTCACGGGTTGGAATAGG	
C1QTNF5	XM_015474686.2	Forward	ATTCTGTCGCGGCATCATCT	83
		Reverse	CCTCTGGTCGTCTGTGAGAG	
PPARD	NM_001083636.1	Forward	GTATGCACATGGTACTCACGC	126
		Reverse	AGCTCTGGCATCATCTGGG	
TGFBI	NM_001205402.1	Forward	TCGCTCCTACCAATGAAGCC	122
		Reverse	GCTGACCAGGATTTCATCGC	
TRIB1	NM_001101105.2	Forward	AGGAAGTTTGTCTTCTCCACCG	136
		Reverse	GAATCTCAGGGCTCACATAGGC	
SIRT4	XM_024977260.1	Forward	GGAAAAGCGAGGACAAACCG	120
		Reverse	CTTCCCACGCATTGAAACGG	
FNIP2	XM_024977606.1	Forward	TGGACCTGAATGAGATTCGCC	148
		Reverse	CTTTTGGCTGGCACTTTCGC	
SLC28A1	XM_010816954.3	Forward	CATCTCTCTCACCTCAGTGGC	128
		Reverse	AAGGCAGTGTATGCATCCCG	

Table S2. The number of sequenced reads and percentage of aligned reads.

Sample ID		Total reads	Total alinged reads	Aligned reads %
Low NFMP	3-1	21,019,444	19,995,159	95.13%
	3-3	20,530,586	19,527,036	95.11%
	10-16	21,546,800	20,492,556	95.11%
High NFMP	3-8	21,212,837	20,296,606	95.68%
	10-8	19,825,469	19,026,640	95.97%
	10-9	19,380,348	18,592,823	95.94%

Table S3. Significant biological process pathways of gene ontology analysis.

GO ID	Term	Count	*P*
GO:0060152	Microtubule-based peroxisome localization	2	0.022
GO:0070268	Cornification	2	0.033
GO:0031397	Negative regulation of protein ubiquitination	3	0.053
GO:0006366	Transcription from RNA polymerase II promoter	5	0.057
GO:0090084	Negative regulation of inclusion body assembly	2	0.065
GO:0008152	Metabolic process	4	0.085
GO:0007059	Chromosome segregation	3	0.094

Table S4. Significant molecular function pathways of gene ontology analysis.

GO ID	Term	Count	*P*
GO:0016887	ATPase activity, coupled	3	< 0.001
GO:0031434	Mitogen-activated protein kinase kinase binding	2	0.048
GO:0004860	Protein kinase inhibitor activity	3	0.068
GO:0003684	Damaged DNA binding	3	0.079
GO:0015279	Store-operated calcium channel activity	2	0.093

Table S5. Significant cellular component pathways of gene ontology analysis.

GO ID	Term	Count	*P*
GO:0072562	Blood microparticle	4	0.048
GO:0005829	Cytosol	18	0.054
GO:0005814	Centriole	4	0.062
GO:0001939	Female pronucleus	2	0.063
